# Supplementary material for: Bioadhesion on Textured Interfaces in the Human Oral Cavity—An In Situ Study
Source: Int J Mol Sci. 2022 Jan 21;23(3):1157. doi: 10.3390/ijms23031157 (PMC8835155; doi:10.3390/ijms23031157)
Supplement: Supplementary file 1 [file ijms-23-01157-s001.zip › ijms-1521400-supplementary.pdf]

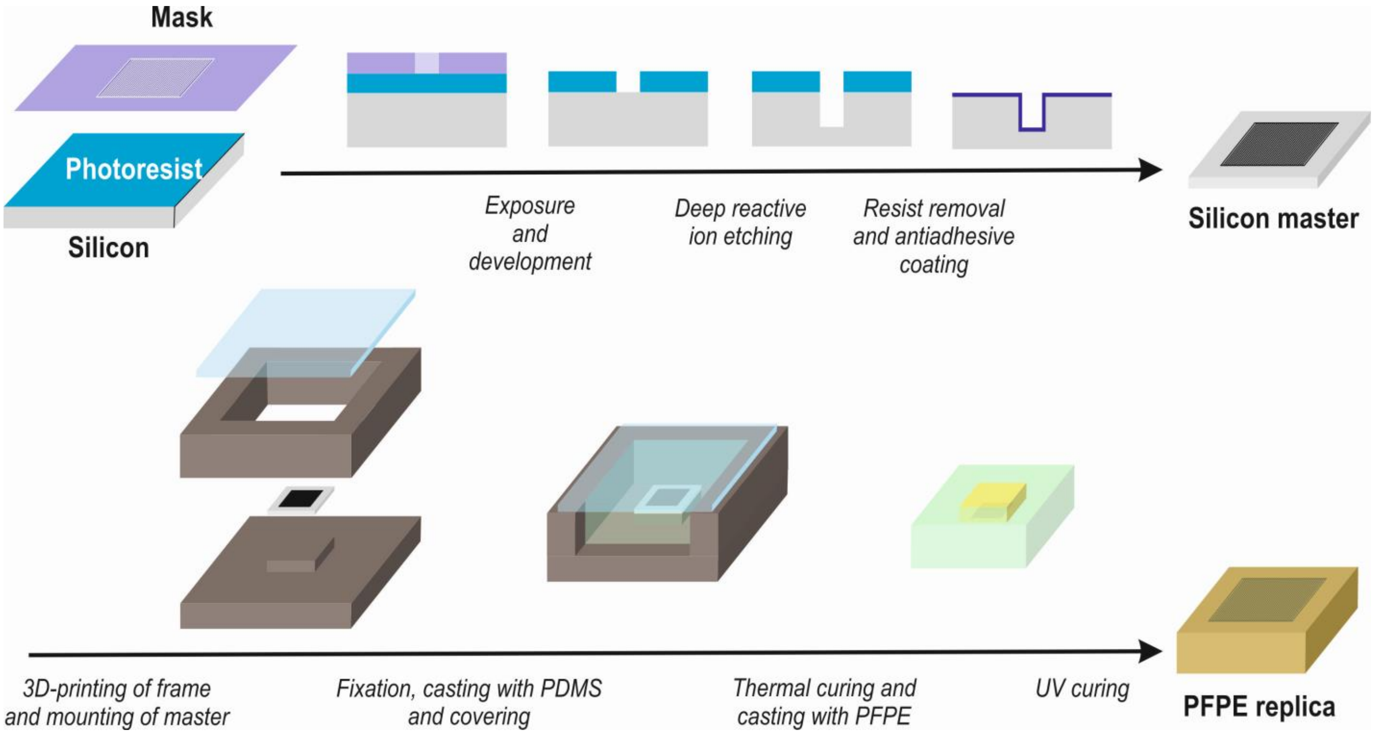

Figure S1: Route of fabrication of structured replica. Silica master structures are manufactured by deep reactive ion etching and transferred into polymeric specimens, which were used for bioadhesion tests.

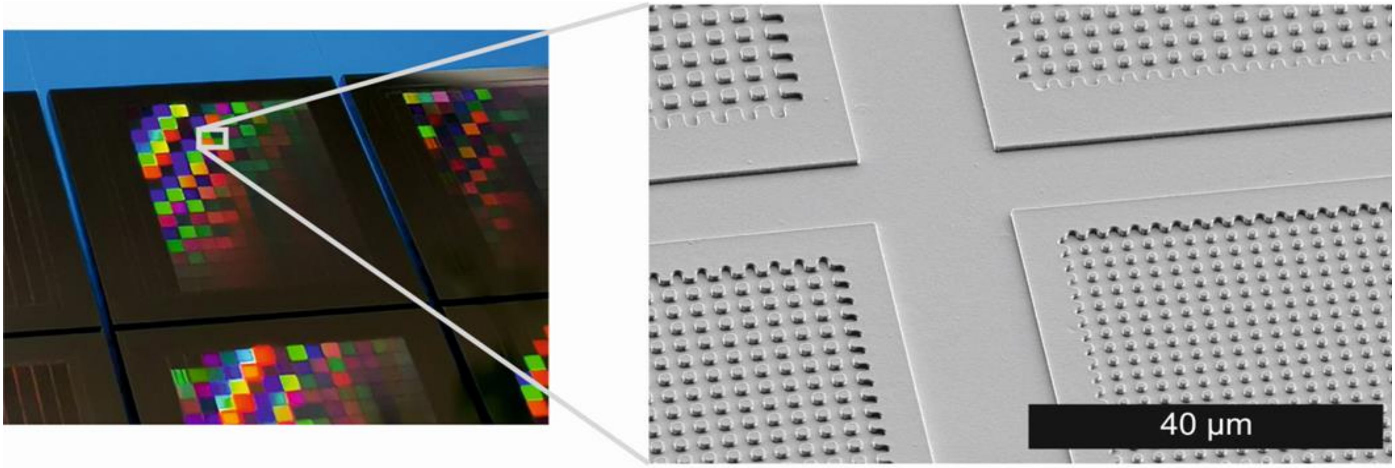

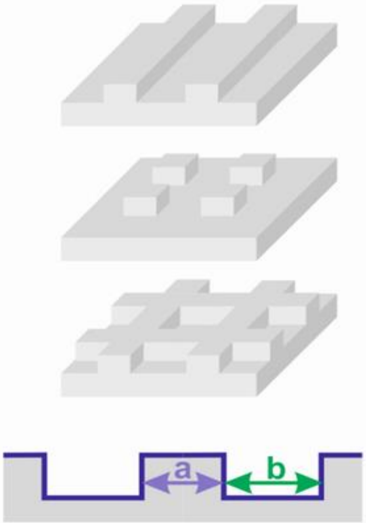

b=x*a		x													
		1.0	1.4	1.8	2.2	2.6	3.0	4.0	5.0	6.0	7.0	8.0	9.0	14.0	19.0	29.0
a [µm]	0.7	0.7	1.0	1.3	1.5	1.8	2.1	2.8	3.5	4.2	4.9	5.6	6.3	9.8	13.3	20.3
	0.8	0.8	1.1	1.4	1.8	2.1	2.4	3.2	4.0	4.8	5.6	6.4	7.2	11.2	15.2	23.2
	1.0	1.0	1.4	1.8	2.2	2.6	3.0	4.0	5.0	6.0	7.0	8.0	9.0	14.0	19.0	29.0
	1.2	1.2	1.7	2.2	2.6	3.1	3.6	4.8	6.0	7.2	8.4	9.6	10.8	16.8	22.8	34.8
	1.5	1.5	2.1	2.7	3.3	3.9	4.5	6.0	7.5	9.0	10.5	12.0	13.5	21.0	28.5	43.5
	2.0	2.0	2.8	3.6	4.4	5.2	6.0	8.0	10.0	12.0	14.0	16.0	18.0	28.0	38.0	58.0
	2.5	2.5	3.5	4.5	5.5	6.5	7.5	10.0	12.5	15.0	17.5	20.0	22.5	35.0	47.5	72.5
	3.0	3.0	4.2	5.4	6.6	7.8	9.0	12.0	15.0	18.0	21.0	24.0	27.0	42.0	57.0	87.0
	3.5	3.5	4.9	6.3	7.7	9.1	10.5	14.0	17.5	21.0	24.5	28.0	31.5	49.0	66.5	101.5
	4.0	4.0	5.6	7.2	8.8	10.4	12.0	16.0	20.0	24.0	28.0	32.0	36.0	56.0	76.0	116.0
	4.5	4.5	6.3	8.1	9.9	11.7	13.5	18.0	22.5	27.0	31.5	36.0	40.5	63.0	85.5	130.5

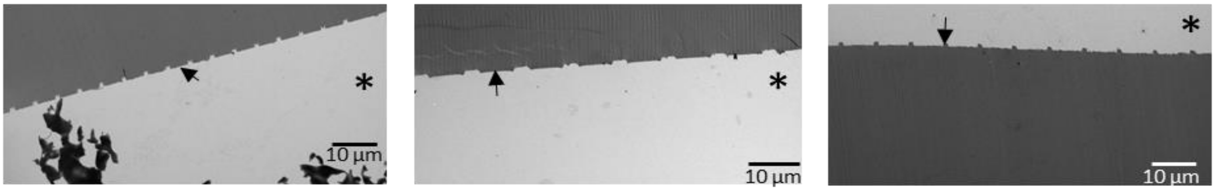

Figure S2: Scheme of gradient organization. Line, pillar and hole gradient structures were created to systematically investigate the impact of structure dimensions on bacterial colonization. Subfields of the whole specimen exhibit increasing structure width, a (i.e. the grid for hole structures) in one direction and increasing spacing, b, (i.e. the cavity width for hole structures) in the orthogonal direction. For each structure width (0.7 to 4.5 µm), the series of increasing spacings result from multiplication with the same series of factors x = 1 to 29. In order to minimize complexity of the presentation of too many data only the orange labeled subfields are evaluated here. Also TEM images show the high regularity of each structure type in a cross-sectional view (lines, holes and pillars from left to right, magnification 1,200-fold). The PFPE side is indicated by an asterisk. Arrowheads point to the recessed linear structures (left), holes (middle) or looming pillars (right). Occasional black dots arose from preparatory steps.

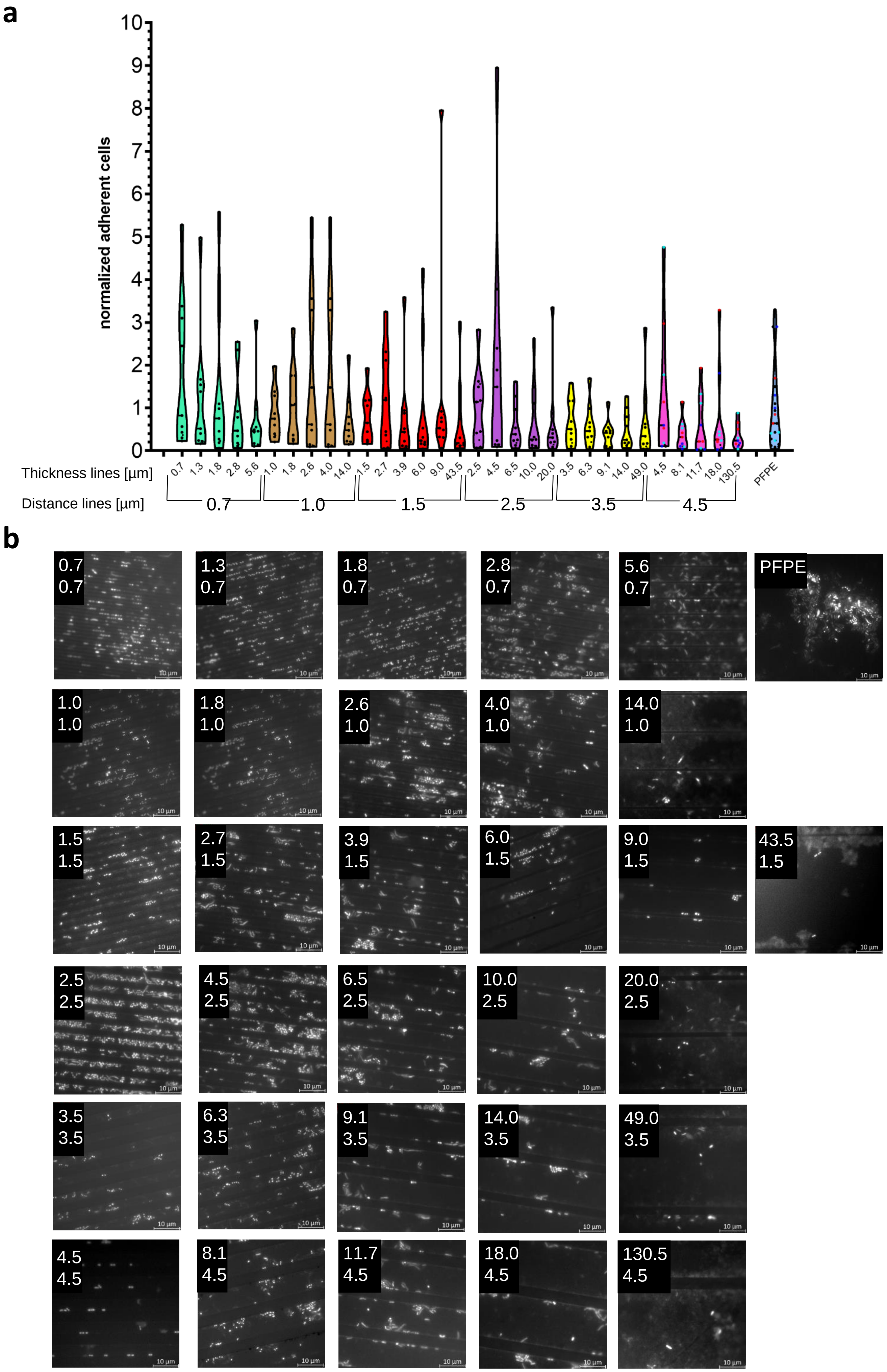
a
normalized adherent cells
0
1
2
3
4
5
6
7
8
9
10
Thickness lines [µm]
0.7 1.3 1.8 2.8 5.6 1.0 1.8 2.6 4.0 14.0 1.5 2.7 3.9 6.0 9.0 43.5 2.5 4.5 6.5 10.0 20.0 3.5 6.3 9.1 14.0 49.0 4.5 8.1 11.7 18.0 130.5 PFPE
Distance lines [µm]
0.7 1.0 1.5 2.5 3.5 4.5
b
0.7 0.7
1.3 0.7
1.8 0.7
2.8 0.7
5.6 0.7
PFPE
1.0 1.0
1.8 1.0
2.6 1.0
4.0 1.0
14.0 1.0
1.5 1.5
2.7 1.5
3.9 1.5
6.0 1.5
9.0 1.5
43.5 1.5
2.5 2.5
4.5 2.5
6.5 2.5
10.0 2.5
20.0 2.5
3.5 3.5
6.3 3.5
9.1 3.5
14.0 3.5
49.0 3.5
4.5 4.5
8.1 4.5
11.7 4.5
18.0 4.5
130.5 4.5
10 µm

Figure S3: Adherence of bacteria to specimens with line structures of different dimensions. (a) Specimens made from PFPE containing linear structures of different dimensions were exposed to the oral cavity for 8 h. Here all analyzed dimensions are shown, a reduced dataset is available in the main manuscript in Fig. 5. Depicted is the number of adherent cells normalized to the adherence on plain PFPE. (b) Representative images of DAPI-stained specimens for the analyzed dimensions. The upper number indicates the thickness of the lines in µm and the lower number the distance of the lines in µm. Microorganisms are mostly detected within the recessed linear structures.

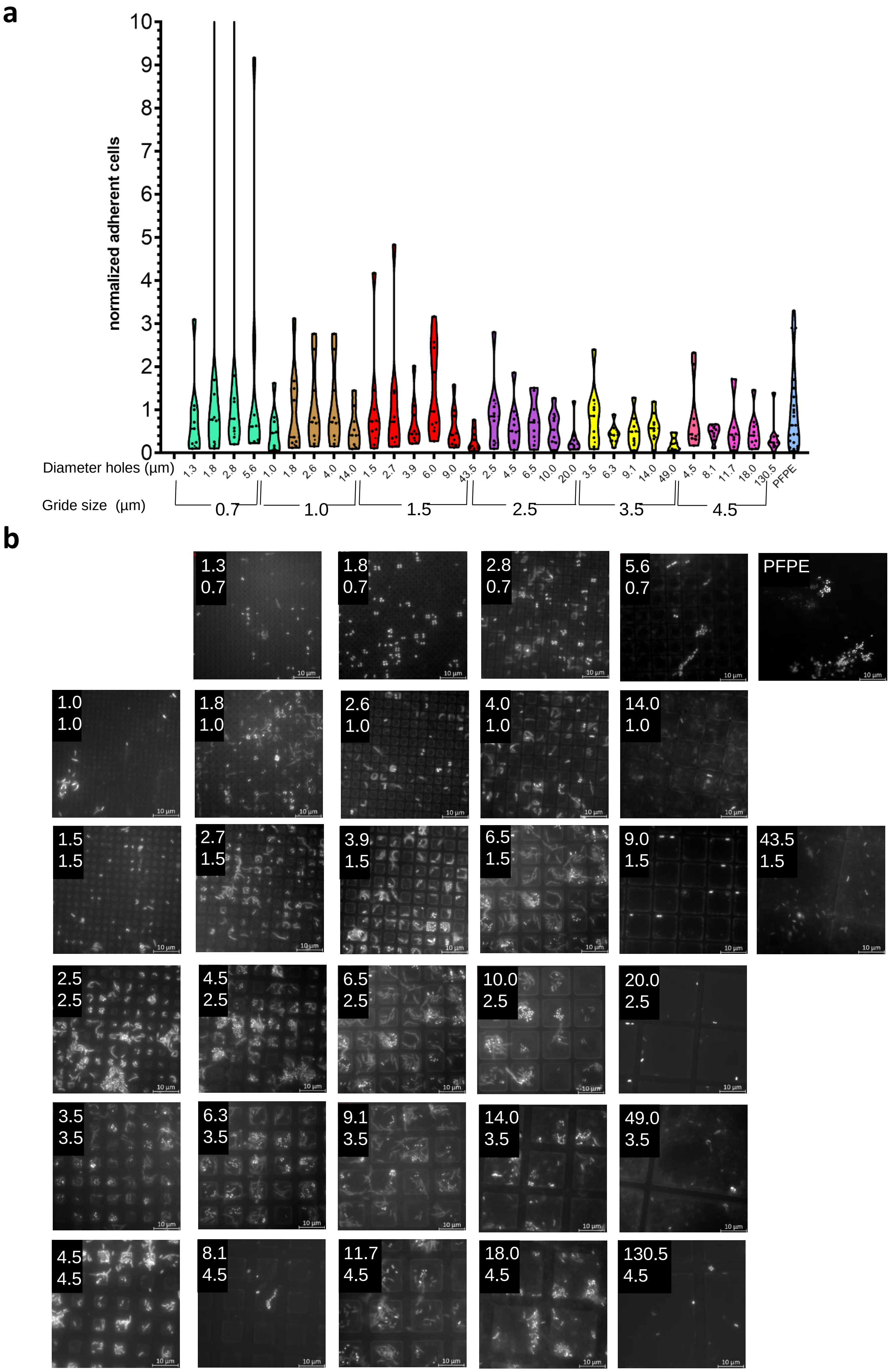
a
normalized adherent cells
0
1
2
3
4
5
6
7
8
9
10
Diameter holes (µm)
1.3 1.8 2.8 5.6 1.0 1.8 2.6 4.0 14.0 1.5 2.7 3.9 6.0 9.0 43.5 2.5 4.5 6.5 10.0 20.0 3.5 6.3 9.1 14.0 49.0 4.5 8.1 11.7 18.0 130.5 PFPE
Gride size (µm)
0.7
1.0
1.5
2.5
3.5
4.5
b
1.3 0.7
1.8 0.7
2.8 0.7
5.6 0.7
PFPE
1.0 1.0
1.8 1.0
2.6 1.0
4.0 1.0
14.0 1.0
1.5 1.5
2.7 1.5
3.9 1.5
6.5 1.5
9.0 1.5
43.5 1.5
2.5 2.5
4.5 2.5
6.5 2.5
10.0 2.5
20.0 2.5
3.5 3.5
6.3 3.5
9.1 3.5
14.0 3.5
49.0 3.5
4.5 4.5
8.1 4.5
11.7 4.5
18.0 4.5
130.5 4.5
10 µm

Figure S4: Adherence of bacteria to specimens with hole structures of different dimensions. (a) Specimens made from PFPE containing hole structures of different dimensions were exposed to the oral cavity for 8 h. Here all analyzed dimensions are shown, a reduced dataset is available in the main manuscript in Fig. 6. Depicted is the number of adherent cells normalized to the adherence on plain PFPE. (b) Representative images of DAPI-stained specimens for the analyzed dimensions. The upper number indicates the diameter of the holes in µm and the lower number the grid size in µm. Microorganisms are mostly detected within the recessed hole-like structures..

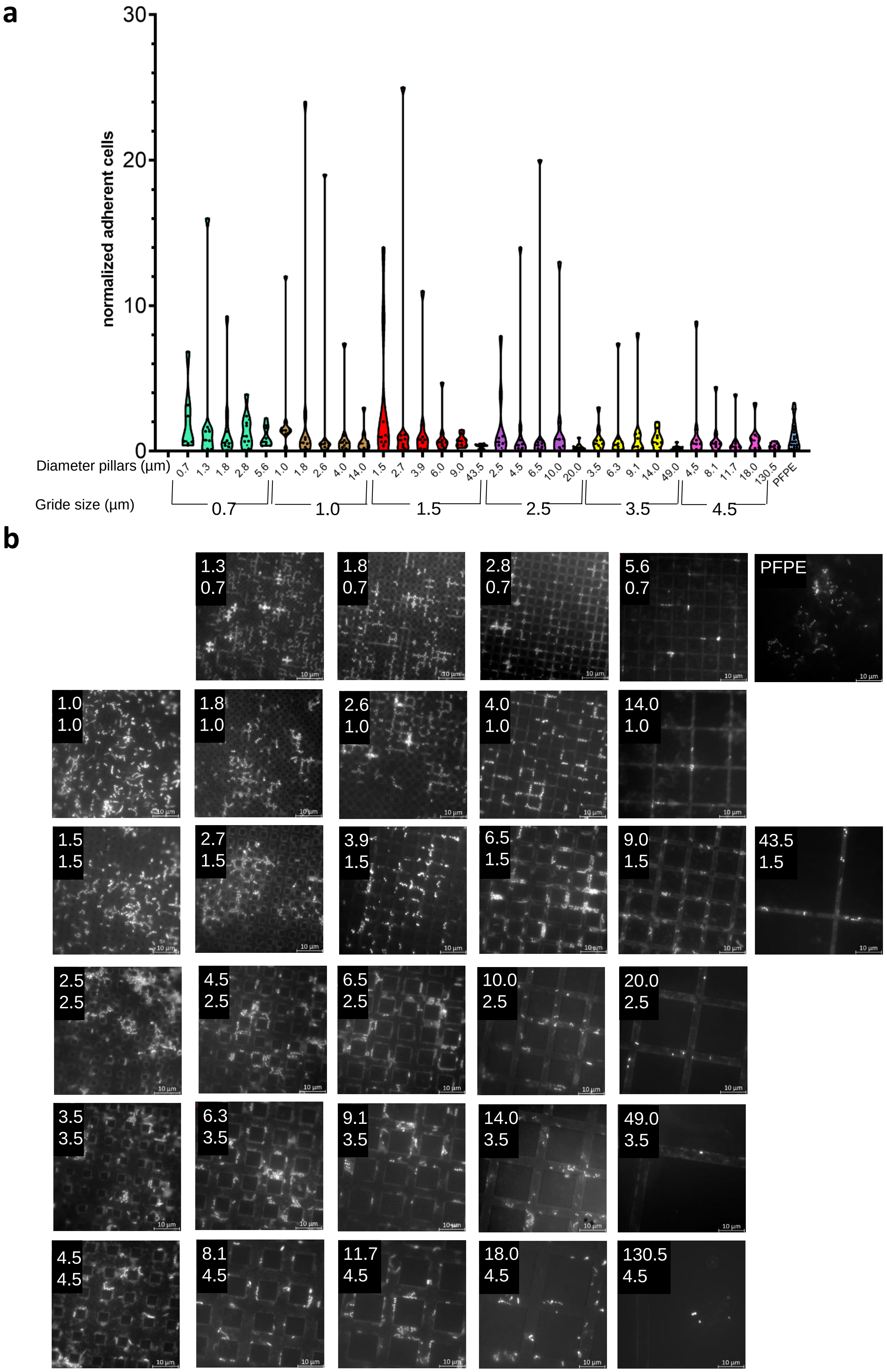
a
normalized adherent cells
0
10
20
30
Diameter pillars (µm)
0.7 1.3 1.8 2.8 5.6 1.0 1.8 2.6 4.0 14.0 1.5 2.7 3.9 6.0 9.0 43.5 2.5 4.5 6.5 10.0 20.0 3.5 6.3 9.1 14.0 49.0 4.5 8.1 11.7 18.0 130.5 PFPE
Gride size (µm)
0.7 1.0 1.5 2.5 3.5 4.5
b
1.3 0.7
1.8 0.7
2.8 0.7
5.6 0.7
PFPE
1.0 1.0
1.8 1.0
2.6 1.0
4.0 1.0
14.0 1.0
1.5 1.5
2.7 1.5
3.9 1.5
6.5 1.5
9.0 1.5
43.5 1.5
2.5 2.5
4.5 2.5
6.5 2.5
10.0 2.5
20.0 2.5
3.5 3.5
6.3 3.5
9.1 3.5
14.0 3.5
49.0 3.5
4.5 4.5
8.1 4.5
11.7 4.5
18.0 4.5
130.5 4.5

Figure S5: Adherence of bacteria to specimens with pillar-like structures of different dimensions. (a) Specimens made from PFPE containing pillar-like structures of different dimensions were exposed to the oral cavity for 8 h. Here all analyzed dimensions are shown, a reduced dataset is available in the main manuscript in Fig. 7. Depicted is the number of adherent cells normalized to the adherence on plain PFPE.(b) Representative images of DAPI-stained specimens for the analyzed dimensions. The upper number indicates the diameter of the pillars in µm and the lower number the grid size in µm. Microorganisms are mostly detected between the pillar-like structures.

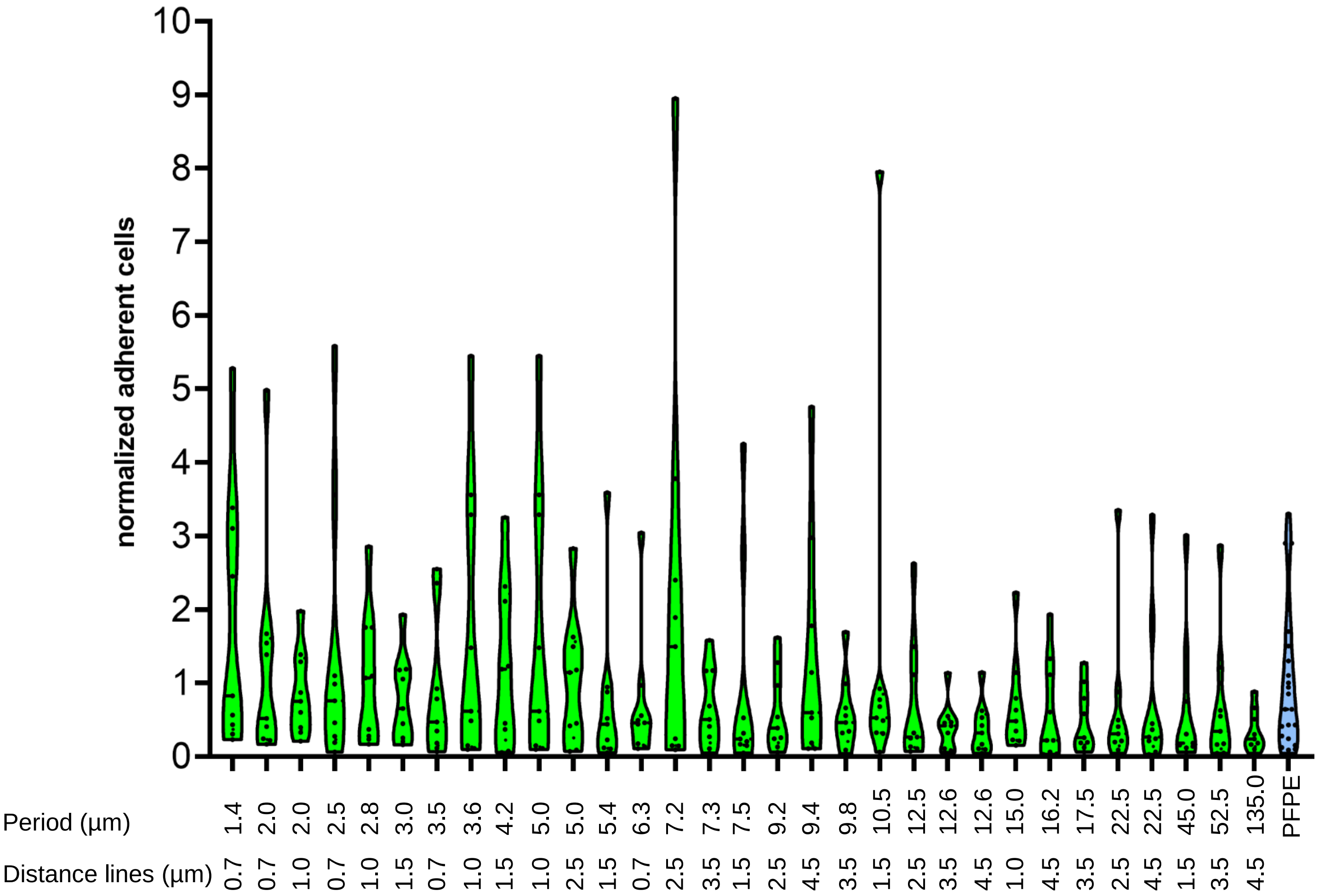

Figure S6: Adherence of bacteria to specimens with line structures of different dimensions ordered by period. (a) Specimens made from PFPE containing linear structures of different dimensions were exposed to the oral cavity for 8 h. Here all analyzed dimensions are shown ordered according to the period. Depicted is the number of adherent cells normalized to the adherence on plain PFPE.

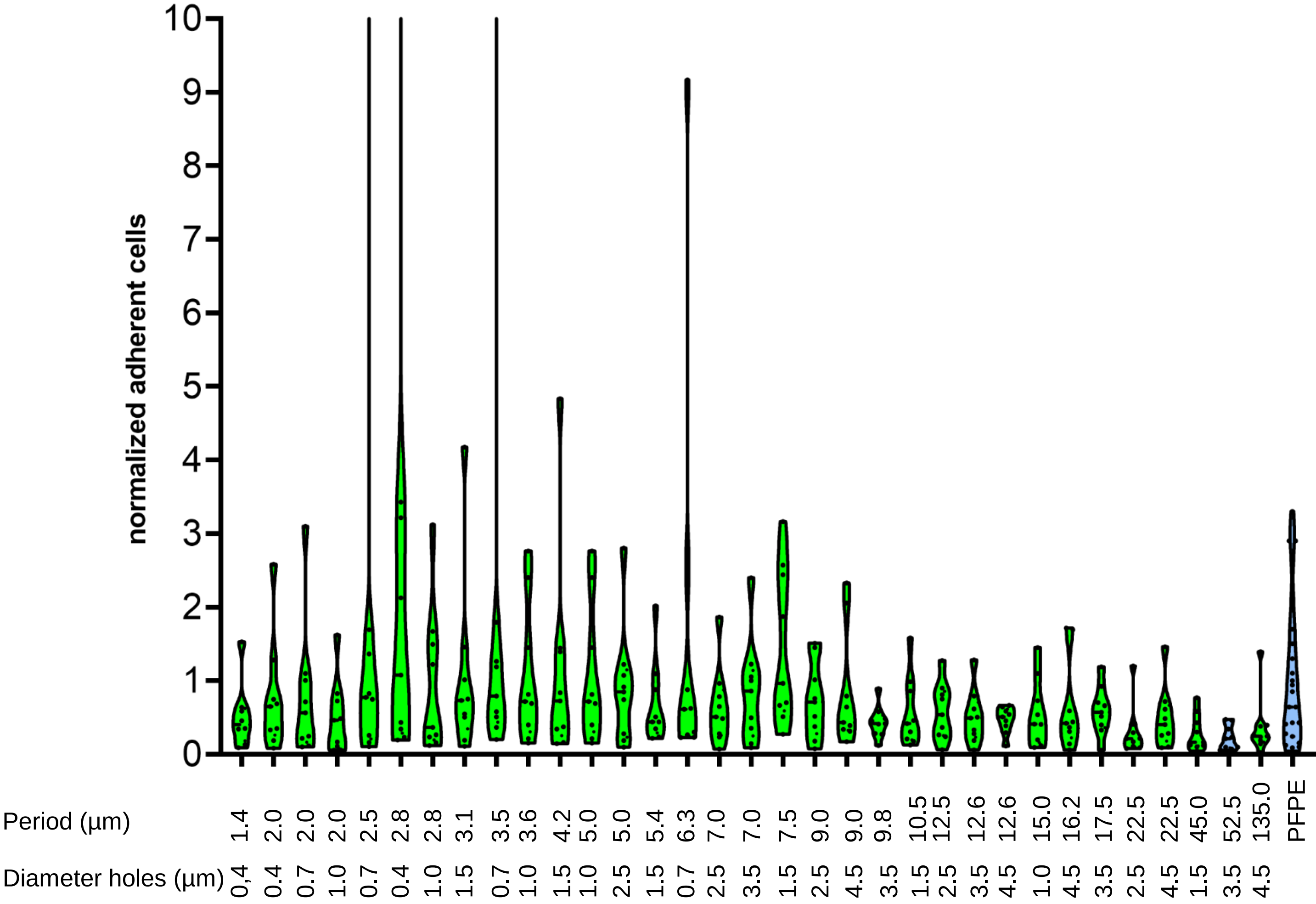

Figure S7: Adherence of bacteria to specimens with hole structures of different dimensions ordered by period. (a) Specimens made from PFPE containing linear structures of different dimensions were exposed to the oral cavity for 8 h. Here all analyzed dimensions are shown ordered according to the period. Depicted is the number of adherent cells normalized to the adherence on plain PFPE.

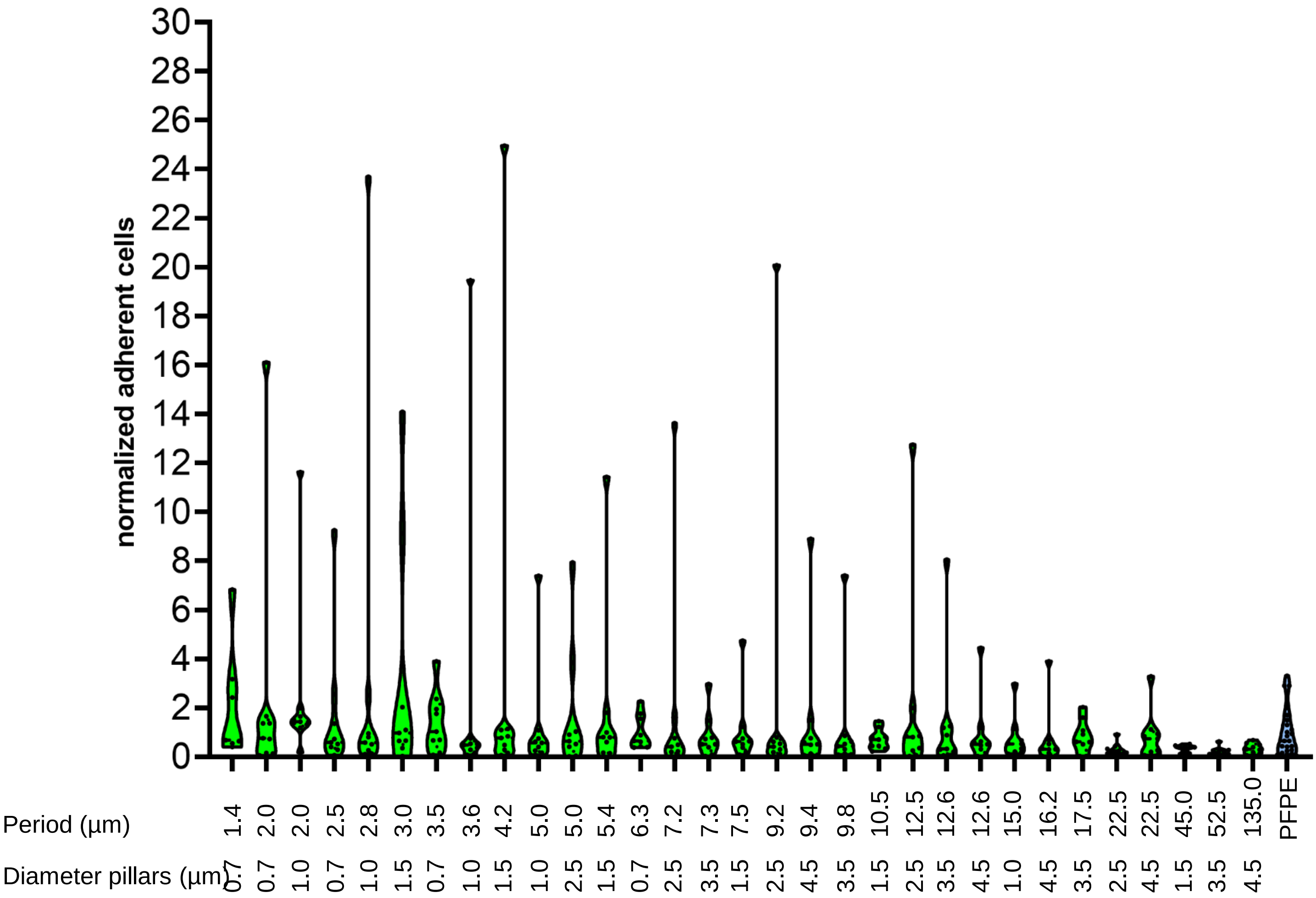

Figure S8: Adherence of bacteria to specimens with pillar structures of different dimensions ordered by period. (a) Specimens made from PFPE containing linear structures of different dimensions were exposed to the oral cavity for 8 h. Here all analyzed dimensions are shown ordered according to the period. Depicted is the number of adherent cells normalized to the adherence on plain PFPE.

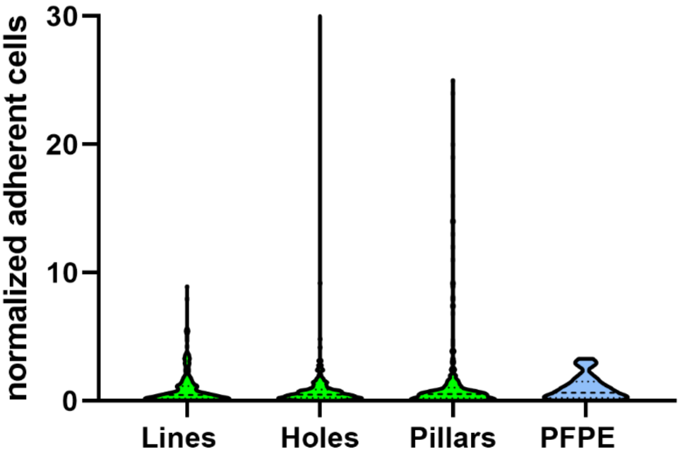

Figure S9: Adherence of bacteria to specimens with structures of different geometries. Specimens made from PFPE containing linear, hole or pillar structures of different dimensions were exposed to the oral cavity for 8 h. Here data for all dimensions are combined from datasets S1-S6.. Depicted is the number of adherent cells normalized to the adherence on plain PFPE. Overall, no significant differences can be observed between the different geometries and plain PFPE.

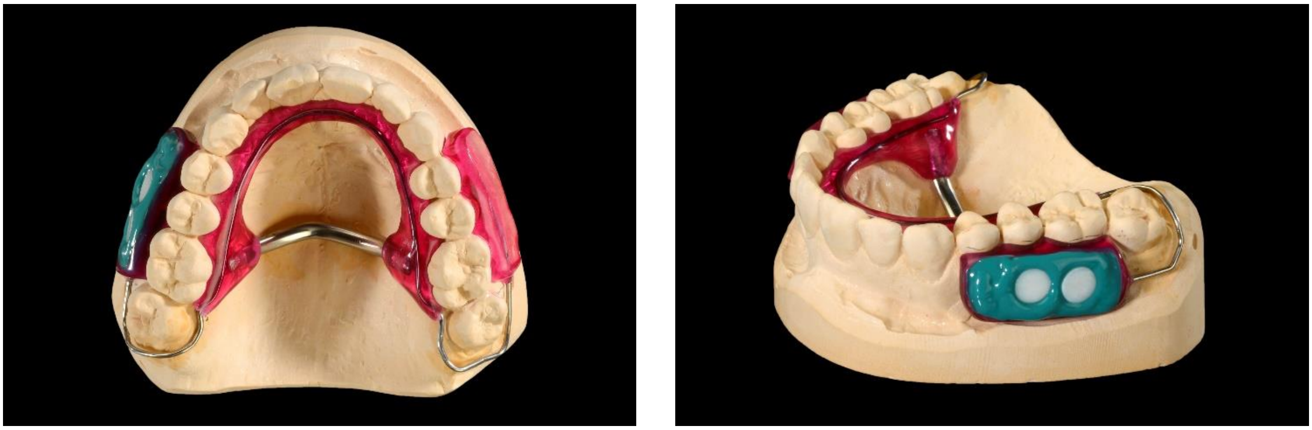

Figure S10: Scheme for the *in situ* experiments: Individual splints were prepared from methacrylate. The specimens were fixed to the splints using polyvenyl siloxane. Then the splints were worn within the oral cavity.
